# Threshold-activated transport stabilizes chaotic populations to steady states

**DOI:** 10.1371/journal.pone.0183251

**Published:** 2017-08-25

**Authors:** Chandrakala Meena, Pranay Deep Rungta, Sudeshna Sinha

**Affiliations:** Department of Physical Sciences, Indian Institute of Science Education and Research Mohali, Knowledge City, SAS Nagar, Punjab, India; Universidad Rey Juan Carlos, SPAIN

## Abstract

We explore Random Scale-Free networks of populations, modelled by chaotic Ricker maps, connected by transport that is triggered when population density in a patch is in excess of a critical threshold level. Our central result is that threshold-activated dispersal leads to stable fixed populations, for a wide range of threshold levels. Further, suppression of chaos is facilitated when the threshold-activated migration is more rapid than the intrinsic population dynamics of a patch. Additionally, networks with large number of nodes open to the environment, readily yield stable steady states. Lastly we demonstrate that in networks with very few open nodes, the degree and betweeness centrality of the node open to the environment has a pronounced influence on control. All qualitative trends are corroborated by quantitative measures, reflecting the efficiency of control, and the width of the steady state window.

## Introduction

Nonlinear systems, describing both natural phenomena as well as human-engineered devices, can give rise to a rich gamut of patterns ranging from fixed points to cycles and chaos. An important manifestion of our understanding of a complex system is the ability to control its dynamics, and so the search for mechanisms that enable a chaotic system to maintain a fixed desired activity has witnessed enormous research attention [1, 2]. In early years the focus was on controlling low-dimensional chaotic systems, and guiding chaotic states to desired target states [3–6]. Efforts then moved on to the arena of lattices modelling extended systems, and the control of spatiotemporal patterns in such systems [7]. With the advent of network science to describe connections between complex sub-systems, the new challenge is to find mechanisms or strategies that are capable of stabilizing these large interactive systems [8].

In this work we consider a network of population patches [9, 10], or “a population of populations” [11]. Now, in analogy with reaction-diffusion processes, diffusive coupling has been very widely studied as a model of connections between population patches, with most models of metapopulation dynamics considering density dependent dispersal [12–16]. However, here we will investigate a different class of coupling, namely threshold-activated transport. The broad scenario underlying this is that each population patch has a critical population density it can support, and when the population in the patch, due to its inherent growth dynamics (which may be chaotic) exceeds this threshold, the excess migrates to neighbouring patches. The neighbouring patch on receiving the migrant population may become over-critical too, triggering further migrations. So this form of coupling is pulsatile and inter-patch transport occurs only when there is excessive build-up of population density in a patch, which may initiate a *cascade of transport events* [6, 17]. Though much less explored, in many situations this form of coupling may be expected to offer a more appropriate description of the connections between spatially distributed population patches.

In this study we will then aim to obtain broad insights on the dynamics of a complex network under threshold-activated transport, through the specific illustrative example of spatially distributed populations connected by threshold-activated migrations. Our principal question will be the following: what is the effect of threshold-activated dispersal on the dynamical patterns emerging in the network, and in particular, can threshold-activated coupling serve to *stabilize the intrinsically chaotic populations in the network to regular behaviour*, such as steady states or regular cycles? In the sections below, we will first discuss details of the nodal dynamics, as well as the salient features of pulsatile transport triggered by threshold mechanisms. We will then go on to demonstrate, through qualitative and quantitative measures, that such threshold-activated connections manage to stabilize chaotic populations to steady states. Further we will explore how the critical threshold that triggers the migration, and the timescales of the nodal dynamics vis-a-vis transport, influences the emergent dynamics.

## Model

Consider a network of *N* sub-systems, characterized by variable *x*_*n*_(*i*) at each node/site *i* (*i* = 1, …*N*) at time instant *n*. Specifically, we study a prototypical map, the Ricker (Exponential) Map, at the local nodes. Such a map has been considered as a reasonably accurate model of population growth of species with non-overlapping generations [18]. It is given by the functional form:

$$\begin{array}{c} x_{n+1}\left(i\right)=f\left(x_{n}\left(i\right)\right)=x_{n}\left(i\right)\exp\left(r\left(1-x_{n}\left(i\right)\right)\right) \end{array}$$ (1)

where *r* is interpreted as an intrinsic growth rate and (dimensionless) *x*_*n*_(*i*) is the population scaled by the carrying capacity at generation *n* at node/site *i*. We consider *r* = 4 in this work, namely, an isolated uncoupled population patch displays *chaotic* behaviour. Note that the results we will subsequently present here, hold qualitatively for a wide class of unimodal nonlinear maps, of which the Ricker map is a specific example.

The coupling in the system is triggered by a threshold mechanisms [6, 19–21]. Namely, the dynamics of node *i* is such that if *x*_*n*+1_(*i*) > *x*_*c*_, the variable is adjusted back to *x*_*c*_ and the “excess” *x*_*n*+1_ − *x*_*c*_ is distributed to the neighbouring patches. The threshold parameter *x*_*c*_ is the critical value the state variable has to exceed in order to initiate threshold-activated coupling. So this class of coupling is *pulsatile*, rather than the more usual continuous coupling forms, as it is triggered *only* when a node exceeds threshold.

Specifically, we study such population patches coupled in a Random Scale-Free network, where the network of underlying connections is constructed via the Barabasi-Albert preferential attachment algorithm, with the number of links of each new node denoted by parameter *m* [22]. The resultant network is characterized by a fat-tailed degree distribution, found widely in nature. The underlying web of connections determines the “neighbours” to which the excess is equi-distributed. Further, certain nodes in the network may be open to the environment, and the excess from such nodes is transported out of the system. Such a scenario will model an open system, and such nodes are analogous to the “open edge of the system”. We denote the fraction of open nodes in the network, that is the number of open nodes scaled by system size *N*, by *f*^*open*^. In this work we also consider closed systems with no nodes open to the environment, where nothing is transported out of the system, i.e. *f*^*open*^ = 0.

So the scenario underlying this is that each population patch has a critical population density *x*_*c*_ it can support, and when the population in the patch, due to its inherent chaotic growth dynamics, exceeds this threshold, the excess population moves to a neighbouring patch. The neighbouring patch on receiving the excess may exceed threshold too. Thus a few over-critical patches may initiate a domino effect, much like an “avalanche” in models of self-organized criticality [23] or cascade of failures in models of coupled map lattices [24]. So the main mechanisms for mitigating excess is through redistribution of excess, which ensures that nodes that are under-critical will absorb some excess population, and through the transport of excess out of the network via the open nodes. All transport activity in the network stops, namely the cascade ceases, when all patches are under the critical value, i.e. all *x*(*i*) < *x*_*c*_.

So there are two natural time-scales here. One time-scale characterizes the chaotic update of the populations at node *i*. The other time scale involves the redistribution of population densities arising from threshold-activated transport. We denote the time interval between chaotic updates, namely the time available for redistribution of excess resulting from threshold-activated transport processes, by *T*_*R*_. This is analogous to the *relaxation time* in models of self-organized criticality, such as the influential sandpile model [23]. *T*_*R*_ then indicates the comparative time-scales of the threshold-activated migration and the intrinsic population dynamics of a patch.

## Results

We have simulated this threshold-coupled scale-free network of populations, under varying threshold levels *x*_*c*_ (0 ≤ *x*_*c*_ ≤ 2). We considered networks with varying number of open nodes, namely systems that have different nodes/sites open to the environment from where the excess population can migrate out of the system. Further, we have studied a range of redistribution times *T*_*R*_, capturing different timescales of migration vis-a-vis population change [25]. With no loss of generality, in the sections below, we will present salient results for Random Scale-Free networks with *m* = 1, and specifically demonstrate, both qualitatively and quantitatively, the stabilization of networks of chaotic populations to steady-states under threshold-activated coupling.

### Emergence of steady states

First, we consider the case of large *T*_*R*_, where the transport processes are fast compared to the population dynamics, or equivalently, the population dynamics of the patch is slow compared to inter-patch migrations. Namely, since the chaotic update is much slower than the transport between nodes, the situation is analogous to the slow driving limit [23]. In such a case, the system has time for many transport events to occur between chaotic updates, and avalanches can die down, i.e. the system is “relaxed” or “under-critical” between the chaotic updates. So when the transport/migration is significantly faster than the population update (namely the time between generations), the system tends to reach a stationary state where all nodal populations are less than critical.

**Fig 1:**
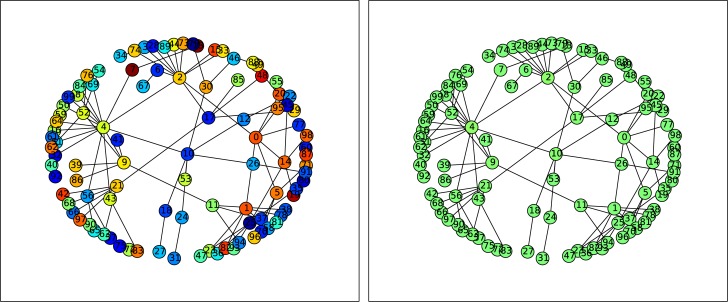
State of the nodes (coded in color) in a Random Scale-Free network of intrinsically chaotic populations under threshold-activated coupling, at different instants of time. Here the steady state value represented by the light green color. The left panel displays the network at initial time, showing the random initial state of the network. The right panel shows the network after 50 time steps, clearly showing that all nodes have evolved to a steady state (as evident from the uniform light green color). Here redistribution time *T*_*R*_ = 5000 and the critical threshold *x*_*c*_ = 0.5, and there is a single node open to the environment.

An illustrative case of the state of the nodes in the network is shown in Fig 1. Without much loss of generality, we display results for a network of size *N* = 100, for a representative large value of redistribution time *T*_*R*_ = 5000. It is clear that *all the nodes in the network gets stabilized to a fixed point*, namely all population patches evolve to a stable steady state.

The next natural question is the influence of the critical threshold *x*_*c*_ on the emergent dynamics, and this will be demonstrated through a series of bifurcation diagrams. Note that in all the bifurcation diagrams presented in this work we will display on the vertical axis the state *x* of a representative site in the network, over several time steps after transience, with respect to threshold *x*_*c*_ which runs along the horizontal axis.

**Fig 2:**
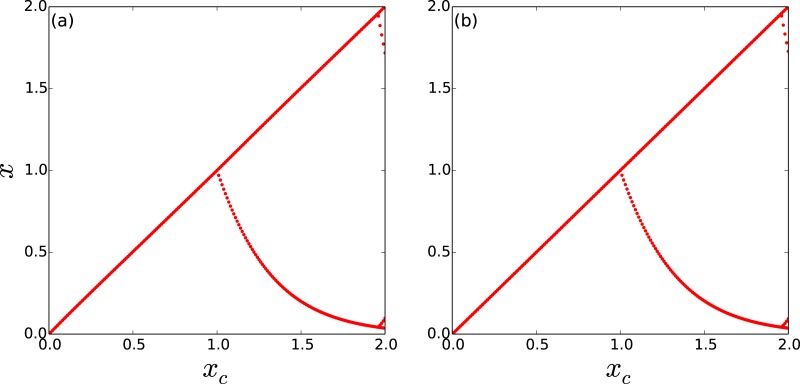
Bifurcation diagrams of the state of a representative node, with respect to critical threshold *x*_*c*_, in a threshold-coupled Random Scale-Free network of intrinsically chaotic populations. Here *T*_*R*_ = 5000 and the network has a single open node, of degree (a) 1 and (b) 15.

It is clearly evident from the bifurcation diagrams in Fig 2 that a *large window of threshold values* (0 ≤ *x*_*c*_ < 1) yield spatiotemporal steady states in the network [26–28]. It is also apparent that the degree of the open node does not affect the emergence of steady states here. Further, for threshold values beyond the window of control to fixed states, one obtains cycles of period 2. Namely for threshold levels 1 < *x*_*c*_ < 2 the populations evolve in regular cycles, where low population densities alternate with a high population densities. This behaviour is reminiscent of the field experiment conducted by Scheffer et al [29] which showed the existence of self-perpetuating stable states alternating between blue-green algae and green algae. We discuss the underlying reason for this behaviour in S1 Appendix, and offer analytical reasons for the range of period-1 and 2 behaviour considering a single threshold-limited map.

So our first result can be summarized as follows: when redistribution time *T*_*R*_ is large and the critical threshold *x*_*c*_ is small, we have very efficient control of networks of chaotic populations to steady states. This suppression of chaos and quick evolution to a stable steady states occurs irrespective of the number of open nodes.

### Influence of the redistribution time and the number of open nodes on the suppression of chaos

Now we focus on the network dynamics when *T*_*R*_ is small, and the time-scales of the nodal population dynamics and the inter-patch transport are comparable. So now there will be nodes that may remain over-critical at the time of the subsequent chaotic update, as the system does not have sufficient time to “relax” between population updates. The network is then akin to a rapidly driven system, with the de-stabilizing effect of the chaotic population dynamics competing with the stabilizing influence of the threshold-activated coupling. So for small *T*_*R*_, the system does not get enough time to relax to under-critical states and so perfect control to steady states may not be achieved.

**Fig 3:**
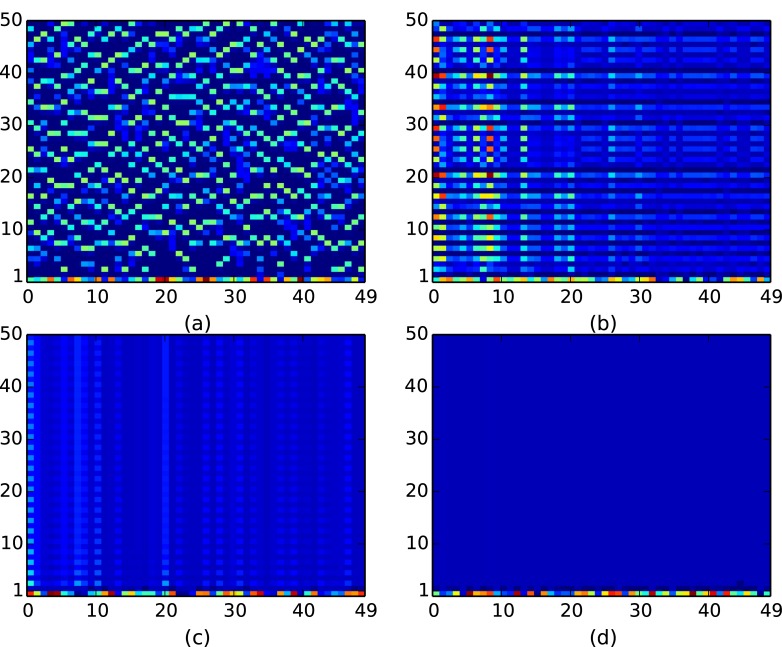
Space-time plots displaying the spatiotemporal behaviour of a Random Scale-Free network of intrinsically chaotic populations. Here time runs along the vertical axis and site index displayed along the horizontal axis. Panel (a) shows the case of uncoupled chaotic populations evolving from a representative random initial state. Panels (b), (c) and (d) show the evolution of the same populations connected through threshold-activated coupling. System size *N* = 50, redistribution time *T*_*R*_ = 50, the critical threshold *x*_*c*_ = 0.5 and the number of open nodes in the network is (b) 1, (c) 10, (d) 30.

**Fig 4:**
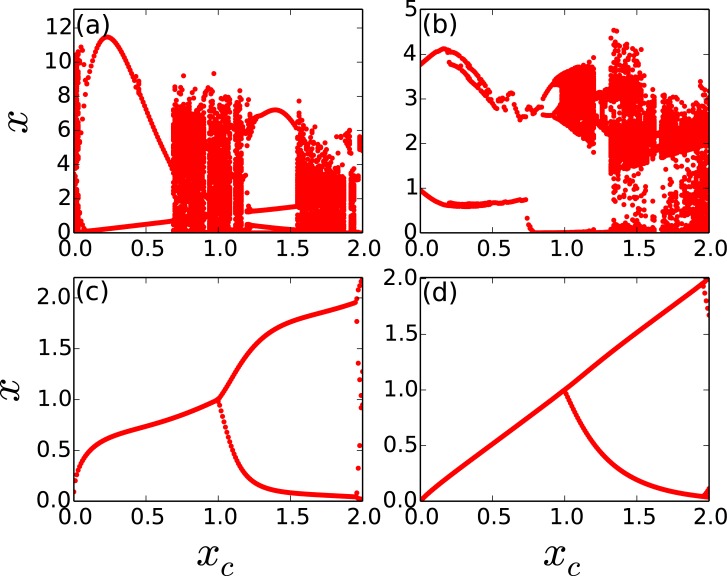
Bifurcation diagrams for one representative node in a threshold-coupled Random Scale-Free network of intrinsically chaotic populations, with respect to critical threshold *x*_*c*_. Here *T*_*R*_ = 50 and the number of open nodes is (a) 1, (b) 10, (c) 30 and (d) 60.

Importantly now, the fraction of open nodes *f*^*open*^ is crucial to chaos suppression. In general, a larger fraction of open nodes facilitates control of the intrinsic chaos of the nodal population dynamics, as the de-stabilizing “excess” is transported out of the system more efficiently. We investigate this dependence, through space-time plots of representative networks with varying number of open nodes and redistribution times (cf. Fig 3), and through bifurcation diagrams of this system with respect to critical threshold *x*_*c*_ (cf. Fig 4).

It is apparent from Fig 3, that when there are enough open nodes, the network relaxes to the steady state even for low redistribution times. Also notice from Fig 3(d) that the system *reaches the steady state very rapidly*, namely within a few time steps, from the random initial state. So more open nodes yields better control of the intrinsic chaos of the nodal population dynamics to fixed populations. This is also corroborated in the bifurcation diagrams displayed in Fig 4, where control to steady states is seen even for low *T*_*R*_, when there are large number of open nodes, vis-a-vis networks with few open nodes. Further contrast this with the dynamics of a system with large *T*_*R*_, shown earlier in Fig 2, where even a *single* open node leads to stable steady states for a large range of threshold values. Similar qualitative trends are also borne out in Random Scale-Free network with *m* = 2, where again more open nodes and longer redistribution times result in better control to fixed population densities.

**Fig 5:**
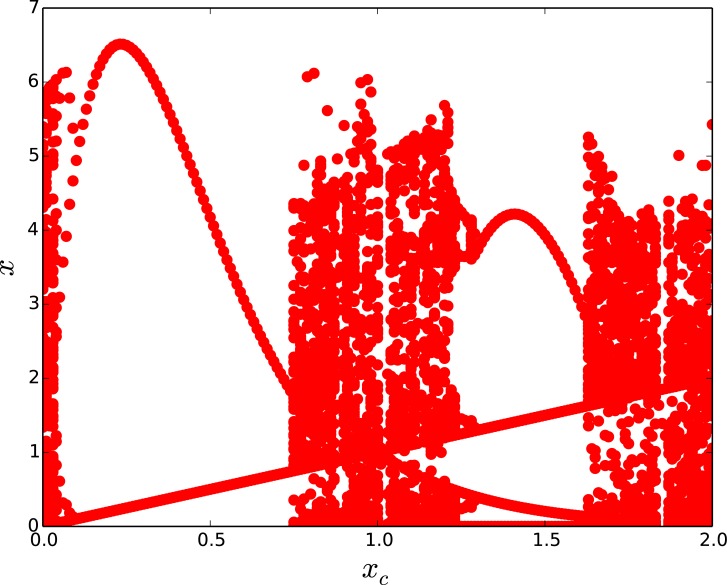
Bifurcation diagram displaying the state of a representative site, for threshold-coupled populations in a Random Scale-Free network. Here *T*_*R*_ = 5000 and there are no open nodes.

As a limiting case, we also studied the spatiotemporal behaviour of threshold-coupled networks without open nodes. Here the network of coupled population patches is a closed system. Again the intrinsic chaos of the populations is suppressed to regular behaviour, for large ranges of threshold values. However, rather than steady states, one now obtains period-2 cycles. This is evident through the bifurcation diagram of a closed network (cf. Fig 5) vis-a-vis networks with at least one open node (cf. Fig 2). Also, note the similarity of the bifurcation diagram of the closed system with that of a system with low *T*_*R*_ and few open nodes. This similarity stems from the underlying fact that in both cases the network cannot relax to completely under-critical states by redistribution of excess between the population updates, either due to paucity of time for redistribution (namely low *T*_*R*_) or due to the absence of open nodes to transport excess out of the system.

Further, we explore the case of networks with very few (typically 1 or 2) open nodes, and study the effect of the degree and betweenness centrality of these open nodes on the control to steady states. Betweenness centrality of a node is given as $b\left(i\right)=\sum_{s,t\in I}\frac{\sigma (s,t|i)}{\sigma (s,t)}$, where *I* is the set of all nodes, *σ*(*s*, *t*) is the number of shortest paths between nodes *s* and *t* and *σ*(*s*, *t*|*i*) is the number of shortest paths passing through the node *i*. We expect the degree and betweenness centrality of the open nodes to play a significant role for the following reason: the main mechanism for mitigating excess is through the transport of excess out of the network via the open nodes. This implies that the emergence of steady states is crucially dependent on the movement of excess occuring at any node in the network to an open edge in *T*_*R*_ steps. So if an open node has more links to other nodes (namely, is of high degree), this would naturally facilitate the transport of excess to it, in parallel, through its many links. Also, an open node with high betweeness centrality implies that the node lies on many shortest paths connecting pairs of nodes. So this too should aid the process, as excess can reach the open node in fewer time steps.

**Fig 6:**
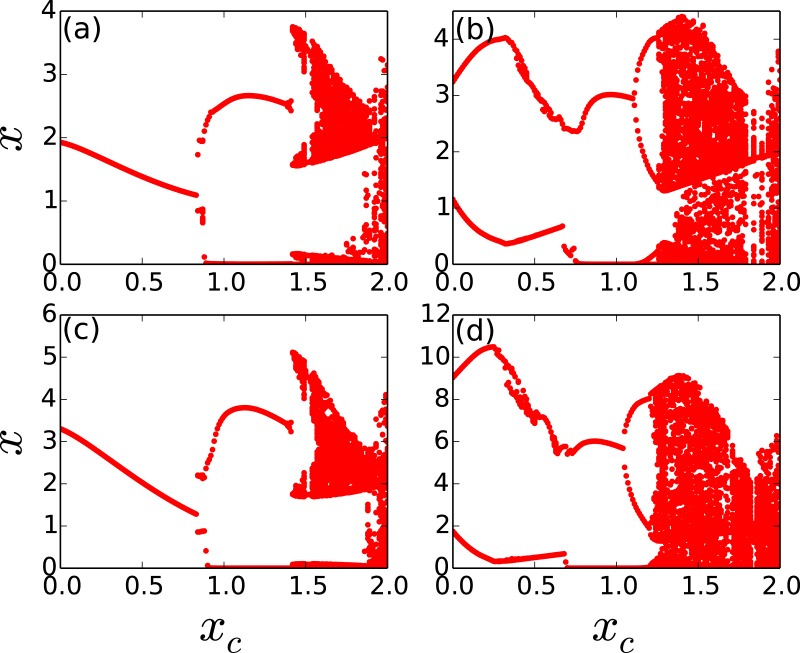
Bifurcation diagrams displaying the state of a representative node, with respect to critical threshold *x*_*c*_, in a threshold-coupled Random Scale-Free network of intrinsically chaotic populations. Here *T*_*R*_ = 500 and there is a single open node, with this open node having (a) the highest betweenness centrality, (b) the lowest betweenness centrality, (c) the highest degree and (d) the lowest degree in the network.

Our expectations above are indeed verified through extensive simulations, where we observe the following: when there are very few open nodes, the degree and betweenness centrality of the open node is important, with the region of control being large when the open node has the high degree/betweenness centrality, and vice versa [30]. This interesting behaviour is clearly seen in the bifurcation diagrams shown in Fig 6a–6d, which demonstrate that the degree and betweeness centrality of the open node has a pronounced influence on control.

### Quantitative measures of the efficiency of chaos suppression

**Fig 7:**
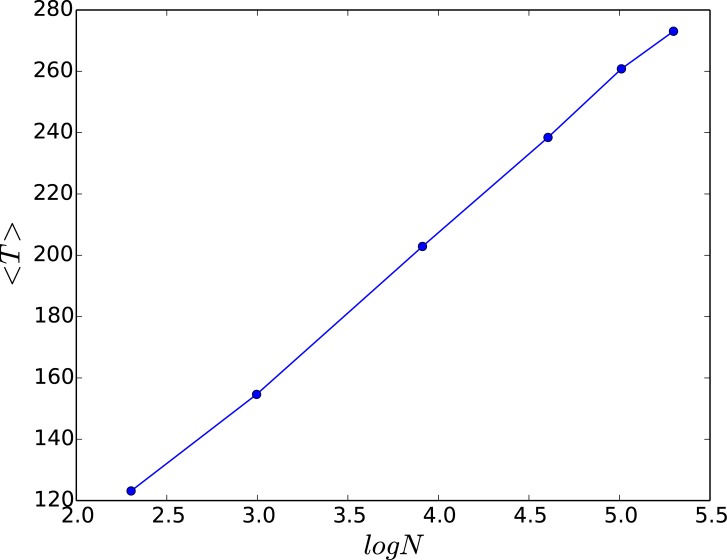
Average redistribution time 〈*T*〉, as a function of the logarithm of the network size *N*. Here 〈*T*〉 is defined as the time taken for all nodes in a system to be under-critical (i.e. *x*_*i*_ < *x*_*c*_, ∀*i*), averaged over a large sample of random initial states and network configurations, the fraction of open nodes in the network is 0.2 and *x*_*c*_ = 0.5.

We now investigate a couple of quantitative measures that provide indicators of the efficiency and robustness of the suppression of chaos in the network. The first quantity is the average redistribution time 〈*T*〉, defined as the time taken for all nodes in a system to be under-critical (i.e. *x*_*i*_ < *x*_*c*_ for all *i*), averaged over a large sample of random initial states and network configurations. So 〈*T*〉 provides a measure of the efficiency of stabilizing the system, and reflects the rate at which the de-stabilizing “excess” is transported out of the network. Fig 7 shows the dependence of 〈*T*〉 on system size *N*. Clearly, while larger networks need longer redistribution times in order to reach steady states, this increase is only logarithmic. This can be rationalized as follows: the average redistribution time needed for all nodes in a system to be under-critical reflects the average time taken by the excess from any over-critical node in the network to reach some open edge. So this should be determined by the diameter of the random scale-free graph, namely the maximum of the shortest path lengths over all pairs of nodes in the network, which scales with network size as ln *N*.

**Fig 8:**
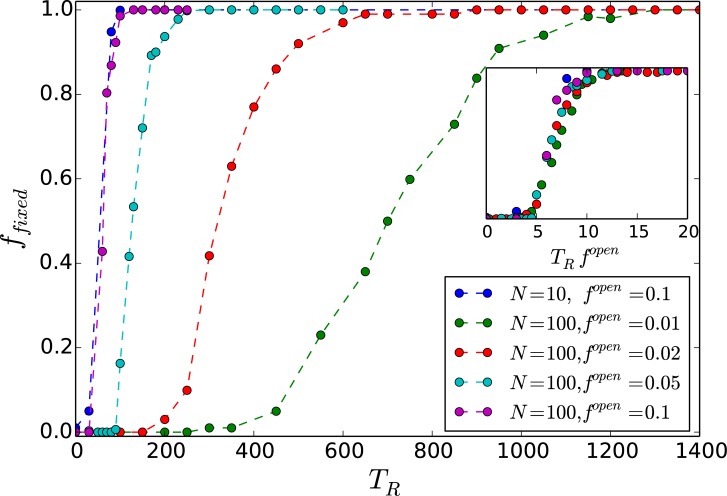
Fraction of nodes in the network that go to steady states, denoted by *f*_*fixed*_, with respect to the redistribution time *T*_*R*_. Here *f*_*fixed*_ is averaged over different network configurations and initial states, *x*_*c*_ = 0.5 and the fraction of open nodes *f*^*open*^ in the network is 0.01, 0.02, 0.05, 0.1 for *N* = 100 (i.e. 1, 2, 5, 10 open nodes in the network respectively) and 0.1 for *N* = 10 (i.e. 1 open node in the network). *Inset*: data collapse indicating the scaling relation *f*_*fixed*_ ∼ *g*(*T*_*R*_ *f*^*open*^).

**Fig 9:**
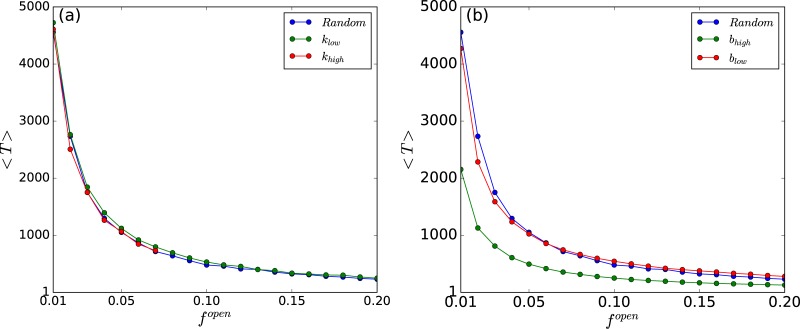
Average redistribution time 〈*T*〉, as a function of the fraction of open nodes in the network *f*^*open*^. Here 〈*T*〉 is defined as the time taken for all nodes in the threshold-coupled Random Scale-Free Network of chaotic populations, to be under-critical (i.e. *x*_*i*_ < *x*_*c*_, ∀*i*), averaged over a large sample of random initial states and network configurations. There are 100 chaotic populations connected via threshold-activated transport in a Random Scale-Free network. In panel (a) the case of open nodes chosen in descending order of degree starting from nodes with the highest *k* (marked as *k*_*high*_) and the case of open nodes chosen in ascending order of degree starting from nodes with the lowest *k* (marked a *k*_*low*_), are displayed. In panel (b) the case of open nodes chosen in descending order of betweeness centrality starting from nodes with the highest *b* (marked as *b*_*high*_) and the case of open nodes chosen in ascending order of betweeness centrality starting from nodes with the lowest *b* (marked a *b*_*low*_), are displayed. In both panels, the case of open nodes chosen at random is also shown for reference.

This is further corroborated by calculating the average fraction of nodes in the network that go to steady states with respect to the redistribution time *T*_*R*_, for networks of different sizes, with varying number of open nodes (cf. Fig 8). Clearly for small systems, with sufficiently high *f*^*open*^, very low *T*_*R*_ can lead to stabilization of all nodes. Importantly, when the fraction of open nodes is very small, the average redistribution time 〈*T*〉 depends sensitively on the betweenness centrality of the open node, and to a lesser extent its degree. Fig 9a and 9b present illustrative results demonstrating this observation.

**Fig 10:**
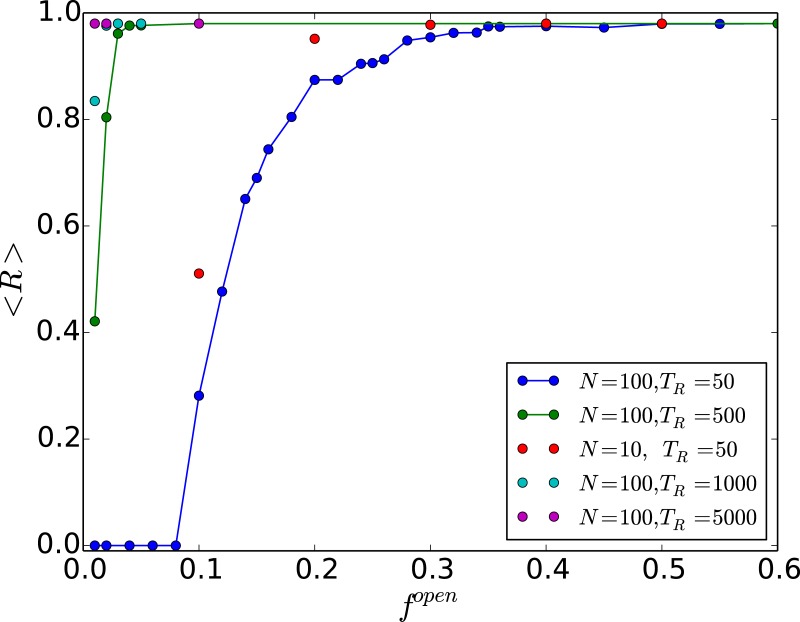
Range of threshold values that yield steady states, 〈*R*〉, as a function of the fraction of open nodes in the network *f*^*open*^. Here 〈*R*〉 is averaged over different network configurations and initial states and the open nodes are randomly chosen. Results from different redistribution times (*T*_*R*_ = 50, 500, 1000, 5000) and system sizes (*N* = 10, 100) are shown.

Next we examine the *range of threshold values yielding steady states*, averaged over a large sample of network configurations and initial states, denoted by 〈*R*〉. Larger 〈*R*〉 implies that steady states will be obtained in a larger window in *x*_*c*_ space, thereby signalling a more robust control. We have explored the dependence of this quantity on redistribution time *T*_*R*_, and also on the fraction of open nodes in the network, denoted by *f*^*open*^. From Fig 10 we see that the steady-state window in *x*_*c*_ rapidly converges to ∼1 (namely, the range 0 ≤ *x*_*c*_ < 1), as the number of open nodes increases. So the window yielding suppression of chaos is almost independent of the number of open nodes, after a sufficiently large fraction of open nodes. Also notice that there is a critical fraction of open nodes $f_{c}^{open}$, after which the network yields a non-zero range of steady states, namely 〈*R*〉 > 0 for $f^{open}>f_{c}^{open}$. We observe that $f_{c}^{open}$ tends to zero as the redistribution time increases and system size decreases, implying that *very few open nodes are necessary in order to lead the network to a steady state* [31].

One can understand these observations by noting that $f_{c}^{open}$ is determined by the time avalable to the system for threshold-activated transport (i.e. *T*_*R*_), and the system size *N*. Now, as mentioned earlier, the average redistribution time needed for all nodes in a system to be under-critical, which reflects the time taken by the excess from any over-critical node in the network to reach some open edge, should scale with the diameter of the random scale-free network. This is known to scale with network size as ln *N*. Further, note that not all nodes are open, and so the probability of reaching an open edge is inversely proportional to *f*^*open*^. This implies that it takes longer to move all the excess to the open node(s) when *f*^*open*^ is smaller. In order to reach a steady state the average time for cascades to cease should be less than the available time *T*_*R*_. So if the available redistribution time *T*_*R*_ is low, and the network size *N* is large, the cumulative excess from all the over-critical nodes in the system will not manage to reach the open edge. So no steady states will emerge (i.e. 〈*R*〉 = 0). However, once *f*^*open*^ > ln *N*/*T*_*R*_, a global steady state will emerge. This offers as estimate of $f_{c}^{open}$. For instance, for *N* = 100 and *T*_*R*_ = 50, this argument suggests that $f_{c}^{open}∼0.09$, which is close to the numerically obtained value. This also implies that for sufficiently large redistribution time, or small enough network size, the system can attain steady state even when there is a *single* open node (i.e. $f_{c}^{open}\to 0$). In fact we can also obtain an estimate for the minimum *T*_*R*_, which we denote as $T_{R}^{min}$, neccessary for allowing the network to reach a steady state with just a single open node, namely $f_{c}^{open}=1/N$. So for networks of size *N* = 100 one obtains $T_{R}^{min}∼N\ln N∼460$, while for networks of size *N* = 10 one obtains $T_{R}^{min}∼23$. This estimate is consistent with the numerical results shown in Fig 10, from where it is clear that for when $T_{R}>T_{R}^{min}$, e.g. for *N* = 10, *T*_*R*_ = 50 and for *N* = 100, *T*_*R*_ = 500, 1000, 5000, 〈*R*〉 is always non-zero, while for *N* = 100, *T*_*R*_ = 50 (i.e. when $T_{R}<T_{R}^{min}$) 〈*R*〉 = 0 for $f^{open}<f_{c}^{open}$, after which there is a transition to non-zero 〈*R*〉.

**Fig 11:**
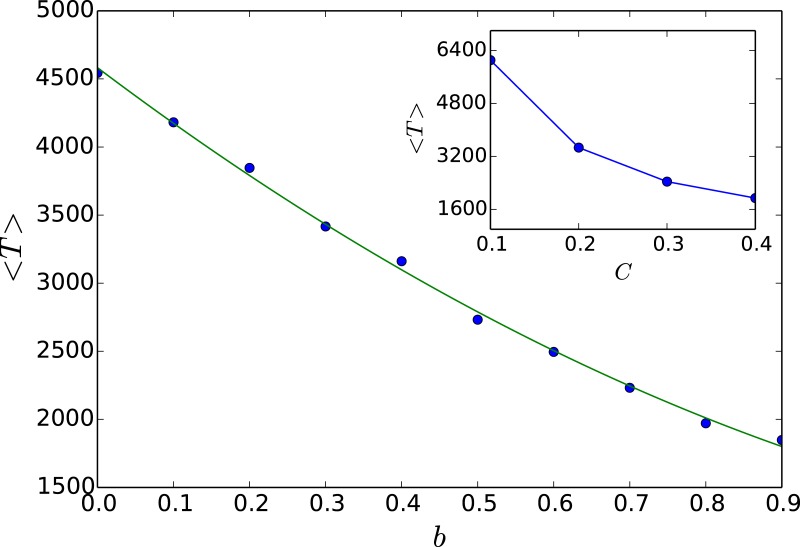
Average redistribution time 〈*T*〉, as a function of the betweeness centrality *b* of the open node. Here 〈*T*〉 is defined as the time taken for all nodes in the threshold-coupled Random Scale-Free Network of chaotic populations, to be under-critical (i.e. *x*_*i*_ < *x*_*c*_, ∀*i*), averaged over a large sample of random initial states and network configurations, in a network with a single open node. The solid curve shows the best quadratic polynomial fit. *Inset*: Average redistribution time 〈*T*〉, as a function of the closeness centrality *C* of the open node.

**Fig 12:**
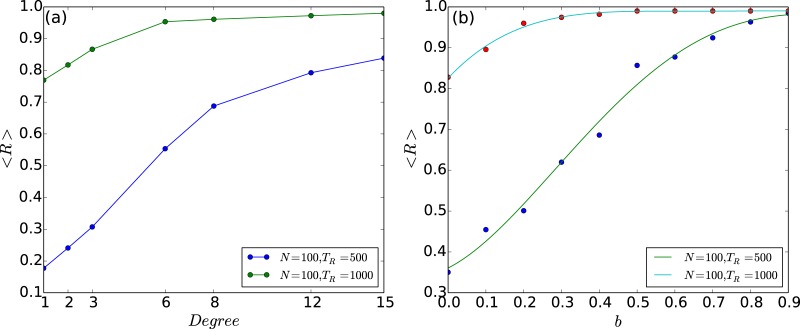
Range of threshold values that yield steady states 〈*R*〉, as a function of the (a) degree *k*, and (b) betweeness centrality *b*, of the open node. Here 〈*R*〉 is averaged over different network configurations and initial states, in a network with a single open node, (with the solid curve showing the best quadratic polynomial fit).

Lastly we explore the scenario of very few open nodes ($f^{open}<<f_{c}^{open}$) in greater depth, through the quantitative measures 〈*R*〉 and 〈*T*〉. In particular, we investigate the limiting case of a *single* open node. Our attempt will be to understand the influence of the degree *k* and betweeness centrality *b* of the open node on the capacity to suppress chaos. We have already observed the significant effect of the betweeness centrality of the open node on the efficiency of control to steady states through bifurcation diagrams in Fig 6. This is now further corroborated quantitatively by the dependence of 〈*R*〉 and 〈*T*〉, displayed in Figs 11 and 12(b). The effect of the degree of the open node is less pronounced, though it also does have a discernable effect on the suppression of chaos. As evident from Fig 12(a), when the open node has a higher degree, it has a higher 〈*R*〉, indicating that open nodes with higher degree yield larger steady state windows.

Finally, note that the different centrality measures are most often strongly correlated and therefore do not offer new insights. For instance, we have also studied the network with respect to open nodes of varying closeness centrality, where closeness centrality is the average length of the shortest path between the node and all other nodes in the graph. We find that qualitatively same broad trends emerge with respect to closeness centrality, as observed for betweenness centrality (cf. Fig 11 and its inset).

These results can be understood intuitively as follows: the emergence of steady states is crucially dependent on the efficacy of the excess being transported out of the network. Namely, excess population from any over-critical node in the network needs to reach an open node within *T*_*R*_ steps. So if an open node has high degree transport of excess is facilitated, as the excess can flow to the node simultaneously through its many links. Further one can rationalize the effect of the betweeness centrality of an open node on the stabilization of the steady state, as betweeness is a measure of centrality in a graph based on shortest paths. If an open node has high betweenness centrality, a large number of shortest paths pass through it. This naturally aids the cascading process, as excess reaches the open node in fewer time steps. The trends expected from these arguments are corroborated in the results from simulations shown in Figs 11 and 12.

## Conclusions

We have explored Random Scale-Free networks of populations under threshold-activated transport. Namely we have a system comprising of many spatially distributed sub-populations connected by migrations triggered by excess population density in a patch. We have simulated this threshold-coupled Random Scale-Free network of populations, under varying threshold levels *x*_*c*_. We considered networks with varying number of open nodes, namely systems that have different nodes/sites open to the environment from where the excess population can migrate out of the system. Further, we have studied a range of redistribution times *T*_*R*_, capturing different timescales of migration vis-a-vis population change.

Our first important observation is as follows: when redistribution time *T*_*R*_ is large and the critical threshold *x*_*c*_ is small (0 ≤ *x*_*c*_ < 1), we have very efficient control of networks of chaotic populations to steady states. This suppression of chaos and quick evolution to a stable steady states occurs irrespective of the number of open nodes. Further, for threshold values beyond the window of control to fixed states, one obtains cycles of period 2. Namely for threshold levels 1 < *x*_*c*_ < 2 the populations evolve in regular cycles, where low population densities alternate with a high population densities. This behaviour is reminiscent of field experiments [29] that show the existence of alternating states. We offer an underlying reason for this behaviour through the analysis of a single threshold-limited map.

For small redistribution time *T*_*R*_, the system does not get enough time to relax to under-critical states and so perfect control to steady states may not be achieved. Importantly, now the number of open nodes is crucial to chaos suppression. We clearly demonstrate that when there are enough open nodes, the network relaxes to the steady state even for low redistribution times. So more open nodes yields better control of the intrinsic chaos of the nodal population dynamics to fixed populations. We corroborate all qualitative observations by quantitative measures such as average redistribution time, defined as the time taken for all nodes in a system to be under-critical, and the range of threshold values yielding steady states.

We also explored the case of networks with very few (typically 1 or 2) open nodes in detail, in order to gauge the effect of the degree and betweenness centrality of these open nodes on the control to steady states. We observed that the degree of the open node does not have significant influence on chaos suppression. However, betweenness centrality of the open node is important, with the region of control being large when the open node has the high betweenness centrality, and vice versa.

The emergence of steady states in this system, not only suggests potential underlying mechanisms for stabilization of intrinsically chaotic populations, but also has bearing on the broad problem of control in complex networks. When a steady state is the desired state of the nodal populations in the network, the threshold mechanism offers a very simple and potent strategy for achieving this, as we have demonstrated clearly. If the aim is to prevent steady states, as may be the case in variants of this model relevant to neuronal dynamics, our results suggest what threshold levels need to be avoided in order to prevent evolution to global fixed points. Note that a large class of control strategies entail complicated algorithms to calculate feedback, and these require knowledge of the global network topology and details of the network dynamics, which are often unknown. Here on the other hand, the nodes respond independently at the local level to a simple threshold limiter condition, requiring knowledge of only the local state at any point in time.

Lastly, interestingly, analogs of this class of coupling have been realized in CMOS circuit implementation using pulse-modulation approach [32, 33]. So some of these results may be of potential interest to the engineering community as well. In the biological context, some experiments have studied similar dynamics in replicate laboratory metapopulations of Drosophila [34]. So our results have the potential to be demonstrated in extensions of such experiments in the future.

In summary, threshold-activated transport yields a very potent coupling form in a network of populations, leading to robust suppression of the intrinsic chaos of the nodal populations on to regular steady states or periodic cycles. So this suggests a mechanism by which chaotic populations can be stabilized rapidly through migrations or dispersals triggered by excess population density in a patch.

## Supporting information

S1 Text Appendix. Analysis of a single population patch under threshold-activated transport.
(PDF)

S1 Fig Appendix. Analysis of a single population patch under threshold-activated transport.
(EPS)

S2 Fig Appendix. Analysis of a single population patch under threshold-activated transport.
(EPS)

S3 Fig Appendix. Analysis of a single population patch under threshold-activated transport.
(EPS)
