# Supplementary material for: Threshold-activated transport stabilizes chaotic populations to steady states
Source: PLoS One. 2017 Aug 25;12(8):e0183251. doi: 10.1371/journal.pone.0183251 (PMC5571948; doi:10.1371/journal.pone.0183251)
Supplement: S1 Text — Analysis of a single population patch under threshold-activated transport. (PDF) [file pone.0183251.s001.pdf]

Appendix : Analysis of a single population patch under threshold-activated transport

We will now analyze the dynamics of a single Ricker map, modelling a single population patch, under threshold-activated transport. Specifically then we have the following scenario: in the dynamical evolution of the system, if the updated state exceeds a critical threshold x_c, it transports the excess out of the system and"re-sets" to level x_c. So the effective map of the dynamics is:

$$x_{n+1} = f(x_n) \quad \text{if} \quad f(x_n) < x_c \tag{1}$$
$$x_{n+1} = x_c \quad \text{if} \quad f(x_n) \geq x_c \tag{2}$$

This is effectively a "beheaded" or "flat-top" map, with the curve lying above $x_{n+1} > x_c$ in the usual Ricker map being "sliced" to x_c (cf. Fig. 1a). The level at which the map is chopped off depends on the threshold x_c. The fixed point solution $x^\star$ occurs at the intersection of this $f(x)$ curve and the 45^0 line, namely $x^\star = x_c$. Remarkably, this fixed point is *super-stable* if the intersection occurs at the "flat top", since $f'(x^\star) = 0$ there.

Clearly, as the threshold increases the intersection of the effective map and the 45^0 line is no longer located at the "flat-top". This is clear for the effective maps for $x_c = 0.5$ vis-a-vis that for $x_c = 1.5$ in Fig. 1a. So $x^\star$ for sufficiently high x_c will no longer be stable (eg. $x_c = 1.5$ will not yield a stable fixed point). So we go on to inspect the second iterate of the effective map, in order to ascertain if a stable period-2 cycle is obtained (cf. Fig. 1b). Now the period-2 cycle solutions occur at the intersection of the $f^2(x)$ curve and the 45^0 line, and again this cycle is stable if and only if the intersection occurs at the "flat top", namely where $f'(x) = 0$. In the illustrative example displayed in Fig. 1b it is clear that for $x_c = 0.5$, where the fixed point is super-stable, the period-2 is also naturally super-stable. Interestingly now, for $x_c = 1.5$, which had an unstable fixed point solution, the period-2 solution is super-stable. So higher x_c also controls the intrinsic chaos. However, instead of a stable steady state, it yields stable periodic behaviour.

Alternately, one can understand the emergence of stable cycles under threshold control as follows: The ergodicity of the system ensures that the system will explore the available phase space fully, and the state variable is thus guaranteed to exceed threshold at some point in time. So one can analyse the dynamics of the effective map starting with the initial state at x_c. Now starting from x_c the dynamics will run as in the usual Ricker population map until $x_{n+1} > x_c$, at which point it is re-set back to x_c and the cycle starts again. So once it exceeds the critical value it is trapped immediately in a stable cycle whose periodicity is determined by the value of the threshold. Further, this allows us to exactly obtain the values of threshold x_c that yield stable fixed points x^* (namely period-1). This is simply the range of x_c for which the first iterate of the Ricker map lies above x_c. In this range $f(x_c) > x_c$. So starting from an initial state x_c, we will be updated in the next iterate to a state greater than x_c, leading to the transport of the excess $f(x) - x_c$ out of the system and the "relaxation" of the system to x_c.

The curves $f_n(x_c)$ as a function of threshold x_c are displayed in Fig. 2. For $n = 0$, $f_0(x_c) = x_c$; for $n = 1$, $f_1(x_c) = x_c \exp(r(1 - x_c))$, and in general $f_n(x_c) = f \circ f_{n-1}(x_c) = f \circ f \circ \ldots f(x_c)$. From the figure it can be clearly seen that in the range of $x_c \in [0 : 1]$, $f(x_c) > x_c$. So if the threshold is in this range, the system will evolve quickly to a steady state at $x^* = x_c$, and transport the excess, namely $f(x_c) - x_c$, out of the system after every update of the population in the patch.

Similarly, it can be seen that $f_2(x_c) = f(f(x_c))$ is larger than x_c (while $f(x_c) < x_c$) in the range of threshold $x_c \in (1, 2]$. So in this range of threshold, we obtain a stable period 2 cycle. Namely, the population at x_c evolves to $f(x_c) < x_c$ which then evolves to $f^2(x_c)$. Since $f^2(x_c) > x_c$, it is mapped back to x_c. Hence a cycle of period 2 arises, with the values of the two points in the cycle being x_c and $f(x_c)$. It can be seen from Fig. 2 that this range is from $x_c \sim 1$ to $x_c \sim 2$. This also corroborates the analysis using effective "flat-top" maps (cf. Fig. 1).

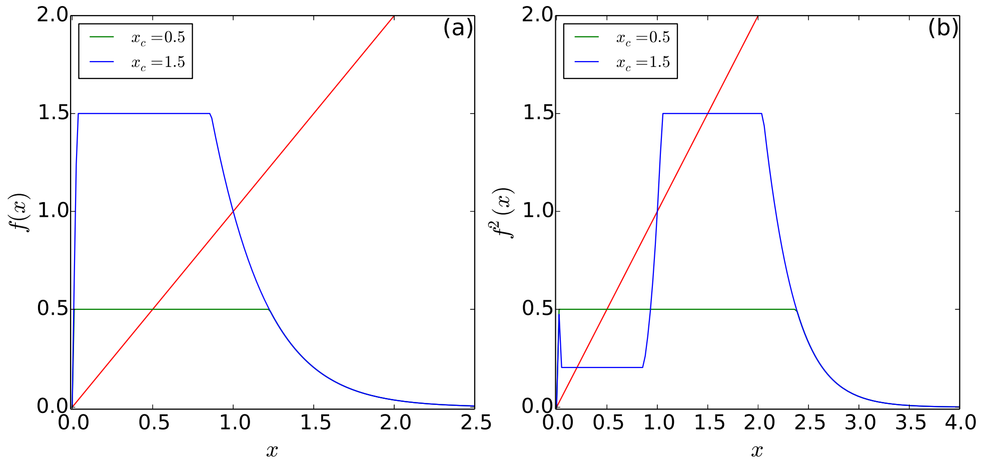

Fig 1. (a) $f(x)$ vs. x and (b) $f^2(x)$ vs. x, for the effective threshold-controlled Ricker map ($r = 4$), for critical threshold levels: $x_c = 0.5$ (green) and $x_c = 1.5$ (blue). The fixed point solution occurs at the intersection of the $f(x)$ curve and the 45^0 line, and is stable if the intersection occurs at the "flat top", namely where $f'(x) = 0$.

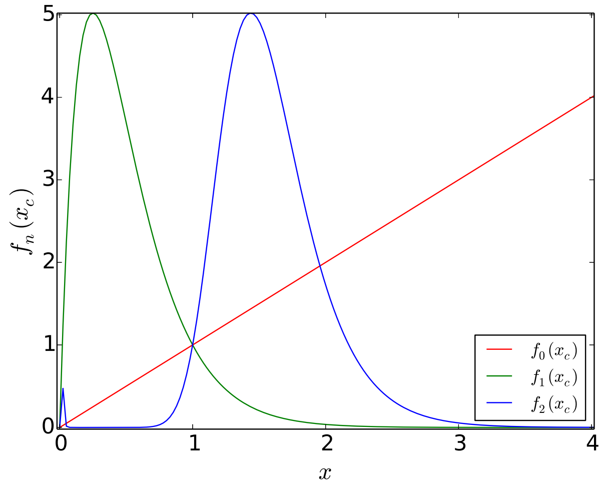

Fig 2. Plot of $f_n(x_c)$ vs x_c. Here $n = 0, 1, 2$, where $f_n(x_c)$ is the nth iterate starting from initial condition $x = x_c$ of the Ricker map with $r = 4$: $f_0(x_c)$ (red), $f_1(x_c)$ (green) and $f_2(x_c)$ (blue).

When there is enough time to relax between chaotic updates (namely T_R is large and/or the number of open nodes is sufficiently high), the collective excess of the network is transported out of the system. This implies that the individual nodes

behave essentially like the “flat-top” map analysed here. This explains why the range of threshold values yielding fixed points and period-2 cycles obtained in networks of threshold-coupled chaotic systems (cf. Fig. 2 of the paper) matches so well with that obtained here (cf. Fig. 3).

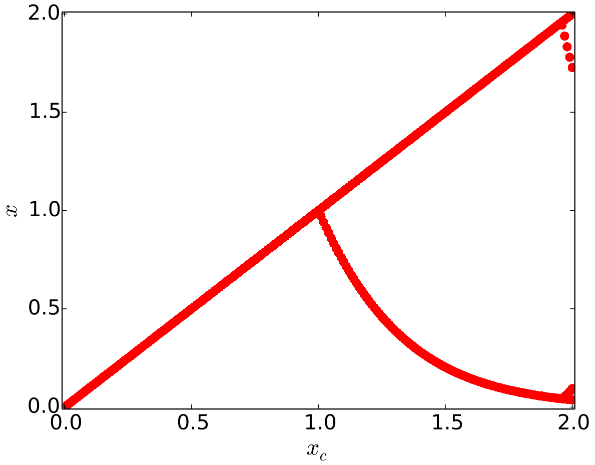

Fig 3. Bifurcation diagram of the threshold-controlled Ricker map (namely, the “flat-top” map), with respect to threshold level x_c.
